# “Imagine if I'm not here, what they're going to do?”—Health‐care access and culturally and linguistically diverse women in prison

**DOI:** 10.1111/hex.12820

**Published:** 2018-09-12

**Authors:** Kelly Watt, Wendy Hu, Parker Magin, Penny Abbott

**Affiliations:** ^1^ School of Medicine University of Western Sydney Penrith NSW Australia; ^2^ School of Medicine and Public Health University of Newcastle Newcastle NSW Australia

**Keywords:** community health worker, cultural and linguistic diversity, health services accessibility, health‐care access, interpreter, primary health care, prisoner, qualitative research

## Abstract

**Background:**

Women in prison have complex medical needs and poorer health status than the general population. Culturally and linguistically diverse (CALD) women in prison, particularly those with limited English proficiency (LEP), have distinct needs and risk additional isolation, discrimination and marginalization when they are in prison.

**Objective:**

We sought to examine how cultural and linguistic diversity, particularly LEP, affects the health‐care experiences of women in prison.

**Design, Setting and Participants:**

We conducted focus groups and semi‐structured qualitative interviews with CALD women and frontline nursing staff in the three female Correctional Centres in New South Wales, Australia.

**Results:**

Participants comprised 30 women in prison and nine nurses. Both women and staff reported communication difficulties as a significant and additional barrier to accessing and receiving health care. For some women with LEP, barriers to care were perceived as discrimination. Fellow prisoners were often utilized as support persons and informal interpreters (“peer interpreters”) in place of formally trained interpreters. While peer interpreters were perceived as useful, potential challenges to their use were vulnerability to coercion, loss of confidentiality, untrained health advice and errors of interpretation.

**Conclusion:**

The persistent use of peer interpreters in prison is complicated by the lack of clearly defined roles, which can include informal peer support roles and lay health advice. These are highly complex roles for which they are unlikely to be adequately trained or supported, despite perceived benefits to their use. Improved understanding and facilitation of health‐related communication could enhance equity of access for CALD women in prison.

## 1 BACKGROUND

In prison, women can experience a profound loss of autonomy with respect to their ability to manage their health.^1^, ^2^, ^3^ Women in prison have complex medical needs and poorer health status than the general population.^4^ However, prison can also provide new opportunities for access to health care.^1^, ^5^

In 2014, 20% of the 683 females in full‐time custody in New South Wales, Australia, spoke a language other than English at home and almost a quarter (23.6%) were born outside Australia, predominantly Vietnam.^4^, ^6^ The number of women in prison continues to rise.^7^

Culturally and linguistically diverse (CALD) women in prison have been described as the “silent” or “forgotten” few,^8^, ^9^, ^10^ referring both to the limited research relating to them^11^ and to their additional isolation, discrimination and marginalization within the prison system.^8^, ^9^, ^10^, ^11^, ^12^, ^13^ Reports on Australian women's prisons describe barriers to communication with staff and other prisoners for women with limited English proficiency (LEP), including a lack of access to interpreters, to information about prison processes and legislative rights, and to programmes and educational opportunities in their own language, and reduced access to religious practices and ministers.^14^ The use of professionally trained interpreters in prisons in Australia and overseas is seen as being suboptimal.^12^, ^15^

Using professionally trained interpreters in health care improves the quality of clinical care more than using ad hoc, or no, interpreters^16^ and significantly reduces the likelihood of errors.^17^ NSW health policy, which applies to the prison health service, mandates that professionally accredited interpreters must be engaged for health‐care communication for all patients who are considered by the health practitioner to not be fluent in English. Additionally, all patients should be informed of their rights to an interpreter, except in medical emergencies or where there is a bilingual health practitioner communicating directly with the patient.^18^

Informal or untrained interpreter use is widespread in health settings despite being problematic.^19^ While patients commonly report a preference for formal interpreters due to the perception of higher quality interpretation, key reasons for use of informal interpreters (often family members) include personal trust and rapport and advocacy.^20^

The unique prison context and added vulnerabilities of patients in prison further complicate the issue of interpreter use, especially as informal interpreters are likely to be fellow prisoners, but there is limited research in this setting. Two qualitative studies of interpreter use in Spanish prisons have reported professional challenges of interpreting in the prison environment^21^ and the ethical difficulties associated with use of fellow prisoners as interpreters.^15^ The use of interpreters in the context of prison health care was not examined, nor did the research focus on the experiences of the prisoners themselves.

In this research, we aimed to explore the complexities of communication and interpreter use for CALD women prisoners accessing prison health care, with a view to improving service access for CALD women in prison. Our research questions were as follows:

1. What is the impact of cultural and language difference on accessing and receiving health care in prison?
2. How do women in prison and health‐care providers manage these impacts?

## 2 METHODS

Using an inductive qualitative approach, we conducted focus groups and individual interviews with CALD women and individual interviews with prison health nurses. This study was undertaken in connection with a larger project into health‐care transitions of women leaving prison.^22^

### 2.1 Setting

Interviews were conducted in three women's prisons in New South Wales, Australia. They ranged from low‐ to high‐security settings, and one was the remand prison for the state. Health care in these correctional centres is delivered under a Board‐governed network under the state health department. It is predominantly a nurse‐led model of care, which requires patients to be triaged by nursing staff prior to seeing medical practitioners.^22^

### 2.2 Sampling

Sampling of the women in prison was purposive for variation in age, cultural background, length of custody, health conditions and health‐care utilization to increase data richness.^23^ We defined CALD women as those women who were born overseas (in a country that did not have English as its primary language) or those who were born in Australia and spoke a language other than English at home. We excluded those born in Australia who spoke English at home to focus on the impact of cultural and linguistic differences.^23^

Potential participants were identified by nursing and custodial staff and by review of a list of current inmates. They were then invited by nursing or custodial staff to meet with the researchers. Peer recruitment also occurred by asking a nurse‐nominated woman to invite other women or friends they felt would be interested in participating. Nurses were purposively sampled to include a variety of patient care roles.

### 2.3 Data collection

KW conducted the focus groups and most of the interviews, with PA conducting 2 interviews and 1 individual interview jointly with KW. As PA was a general practitioner at one of the research sites, she did not conduct any staff interviews or interviews with women to whom she had provided health care.

Interview questions covered how cultural and linguistic diversity affects prison life and health‐care delivery, peer and formal interpreter use in prison and the women's experiences of health care in prison. We defined a peer interpreter as a fellow prisoner from the same cultural background who spoke more English than those they were assisting, but who was untrained in interpreting. A formal interpreter was defined as an interpreter from outside the prison with professional qualifications and training in medical interpreting, used via phone or face‐to‐face.

We obtained written informed consent after explaining via the participants’ chosen interpreter, offering the women participant information and consent forms in English or translated in the woman's language. Formal interpreters were offered to all women. To ensure the credibility of the data when informal interpreters were used, we explicitly explored the interpreter's perspectives on the interview topics during the focus groups to identify any points of divergence from non‐interpreted interviews.^24^ In the focus groups, at times, two or three people acted as informal interpreters for the other women, giving multiple points of interpretation. We also used communication techniques to foster mutual understanding by avoiding multicomponent questions, beginning with open‐ended questions and progressing to more specific queries (“the funnel approach”)^25^ and checking the interviewer's understanding of the response.^26^

Interviews and focus groups were audiotaped and spoken English transcribed verbatim. Two women also provided written data during the focus groups, which they explained was a summary of issues they had experienced with the health service. Focus groups containing background discussion in Vietnamese (the only language spoken other than English in the focus groups) were professionally translated and transcribed into English. These transcripts were then discussed with a bilingual general practitioner who acted as a cultural advisor. Both methods of translation aimed to provide cultural context to the data and to reduce the risk of distortion of the results through translation.^24^, ^25^, ^27^, ^28^

### 2.4 Ethical issues

Transcripts were deidentified and stored securely. Any urgent or serious clinical issues that had not previously been addressed arising from the interviews were passed on to health staff, with the participant's permission, for follow‐up through formal routes. Interviews were conducted in private health clinic rooms, in prison visitation rooms or in privacy in the prison cottage dwellings. Guards and health staff were present nearby but could not hear or see the interview. Given the constraints of prison access, these were considered the most neutral space available.^25^, ^27^ Interviews with the nurses took place in private rooms in the health clinic. A $10 AUD payment was made into each woman participant's in‐prison account in keeping with usual research practice in NSW prisons.

### 2.5 Data analysis

Thematic data analysis was undertaken,^29^ facilitated by the use of NVivo software (version 9, QSR). Transcripts were initially open‐coded by KW and then refined. During this stage, PA and WH independently analysed a selection of transcripts and emergent ideas and concepts were iteratively discussed and tested by returning to the data to develop preliminary themes. Data from women and nurse participant groups were initially analysed separately and then compared across and between groups to provide different perspectives and arrive at the final themes. Memo writing was used extensively throughout the process to provide an audit trail.

## 3 RESULTS

Box 1 provides a description of the women participants (of which there were 30 in total) including estimation of women's language ability using the US Census‐LEP item descriptions.^30^ Women participants who spoke English well commonly reported being peer interpreters for others. Peer interpreters assisted in all three focus groups and one individual interview. Box 2 provides a segment of a focus group interview as an example. One individual interviewee chose to utilize a formally trained interpreter. All other individual interviews were conducted in English without interpretation.

Nine nurses participated in this study. The nurses were all female, reflecting the staff profile, and had worked in the prison health service for between one and 15 years. They had a variety of health‐care roles, namely primary health, mental health, chronic care, public health and women's health, and four worked across more than one prison.

Interviews with women in prison lasted 30‐100 minutes with a median duration of 60 minutes and focus groups lasted 40‐80 minutes. Interviews with nurses lasted 30‐70 minutes.

There were two major themes, which related to the impact of cultural and linguistic difference on prison life and health‐care access, and to health‐care communication and the use of interpreters. These themes and subthemes are presented below with illustrative quotes.

Box 1 **Description of women participants**

1

**Table:** 

Total number = 30
3 focus groups—4‐6 participants each
14 individual interviews
Age
20‐75 years
Time in prison
2 months to 10 years
Background
Vietnamese
Chinese
Lebanese
South American
European
Time in Australia
0 (taken to prison immediately on arrival to Australia) to 20 years
Language ability using the US Census‐LEP item descriptions^34^
Focus groups 1 and 2
All Vietnamese speaking women apart from one Middle Eastern female
Majority of women spoke English “a little” to “not at all” with 3 women speaking English “well” and acting as peer interpreters for majority of interview
Focus group 3
Mixture of Asian and European language backgrounds
Spoke English “well” to “very well” and required minimal peer interpretation
Individual interviews
Two participants in individual interviews spoke English “a little”’ and used interpreters
Five participants fluent with English as their primary language
Seven spoke English “well” to “very well”
Interview length
Individual interviews—30‐100 minutes with a median duration of 60 minutes
Focus groups—40‐80 minutes

Box 2 **English and Vietnamese transcription example**

1 Q = interviewer

PI = peer interpreter

VS = Vietnamese speaker

Professionally translated sections in **bold**

Q: And do you feel comfortable using an interpreter on the phone?

**PI: Each of you, if an interpreter is available on the phone do you feel comfortable with that?**

**VS1: Yes. Yes.**

PI: Yes. They help.

Q: Good.

PI: Better than not know anything.

Q: Absolutely. Yeah.

**VS2: But you should say that you have to wait a long time for an interpreter.**

**VS3: But each time they call for an interpreter it takes a long time.**

PI: If they call, they contact interpreter Vietnamese but wait so long.

Q: It takes a long time.

PI: It takes a long time.

Q: And are the nurses happy to call an interpreter for you?

PI: Yeah they happy because they want understand what they say of course they happy.

Q: do they ask if you would like an interpreter?

**PI: Do they ask, do they ever ask you old lady if you need an interpreter or anything?**

**V1: (Inaudible)… in jail (inaudible)… don't know anything.**

PI: She always drag me along.

Q: Oh [laughs] yep.

**PI: But if I don't interpret for you old lady. Hah?**

**Do you look for someone to interpret for you old lady? Let's say I am not available then there's her, (inaudible)…**

**VS1: Oh well… but if I get ask something and one is not available then I look for someone else, ask for someone else to help interpret because I do not speak English.**

PI: If I'm working if someone available outside the compound, she call that person.

Q: Does that work? And would you always prefer to have someone from the inside or would you prefer a phone interpreter?

**PI: Now do you prefer someone, like someone in here to interpret for you or do you want someone to interpret for you on the phone?**

**VS1: You tell her that for important issues, our own issues then you still need an [formal] interpreter especially. But let's say to go up here to ask something there to ask something it's standard then you can get friends to help. But for those… those which you need to talk about personal things… then you need to have an interpreter.**

PI: For her serious appointment or whatever she want to do, she need a proper interpreter, just for regular or small, mini things just ask some people available to help her.

**VS1: For example you need to see a doctor, a specialist then you need an interpreter, one who specialises in a specialist area; and if you go to court then you need an interpreter who specialises in working in court. Not everyone can interpret, it wouldn't be accurate. With an interpreter it's clear that's what you need. When you go to court for your case, you need the interpreter to be ah… (inaudible)…**

**PI: For that case.**

**VS1: You know? And if it is for a specialist doctor for your breasts, you ass, your (inaudible)…**

**(Laughter)**

**PI: You describe…**

**VS1: Thingy together like that then it's accurate and then it's correct. But if your problem is in your breast and you call for an interpreter for the ass then it won't work, it'd useless!**

PI: If she needs someone to translate for her, like if she got a lawyer translate for her can't use the one that doing better to translate for her.

**VS1: Correct? That's all I know.**

**VS1: Correct? Is that right dear?**

**PI: No.**

**VS1: Why not?**

**PI: The interpreter just say the meaning… and not… ah mm… whatever you ask like you ask me, I interpret that is I say whatever you want to say. Whatever the other person say I interpret that back to you. It's not like you say the breast or this and that. It's not like that.**

**VS1: Well yes I agree with that. I agree that's the case. But the, the area of expertise it's… it's… (clicks her tongue) what I mean to say is let's say ah… yes you answer, say whatever I want to ask**

**PI: But old lady, for specialised area if I am competent in Vietnamese then… I would interpret as exactly as what people say. You don't need a specialised interpreter.**

**VS1: There are specialised terms dear.**

**PI: No. If the interpreter's Vietnamese is really competent…**

**VS1: When you interpret… and… and if you don't know the specialised terms then how do you interpret. Correct? For specialised areas.**

**PI: But they are interpreters they don't sit there to explain things…**

**VS1: Well yes interpret. But the interpreter needs to specialise too. Specialise in what industry and what industry.**

**(laughter)**

**VS1: Yeah. Now let's say that person is competent in ah… this industry and someone else is competent in, specialises in another industry in interpreting. It's not as if you can interpret in whatever situation you want. Then you don't interpret correctly, they don't understand those meanings, in specialised area. And specialised in ah… ah… the breasts let's say and you send an interpreter who speak in another matter then he won't know what the special term for breast is.**

**VS2: Wait let her say a few words back to her or else she will think that we are saying something bad about her.**

Q: So you have to have a different person. Would you mind translating?

PI: She say, like when you have a translation, like it has to be in a certain group, like if you – what do you say?

PI: Have the lawyer for the law.

Q: So they need to know about law.

PI: But for me, like because translation because when you're doing a translation, it's meaning a translation. This don't have to be like lawyer, dentist and all the other parts.

Q: And so do you feel that if you had the phone interpreter, do they know all the medical words and things like that?

PI: No.

Q: So the phone interpreters, do they not know the ‐ ‐ ‐

PI: Sometimes it's just basic, they translate on a base for you to understand easier.

Q: Oh sure, yeah, and is that better to have the basic translation?

PI: Yes.

Q: Rather than all their medical words. Okay.

PI: Even you say a medical word, we wouldn't understand either.

### 3.1 The impact of cultural and language difference on prison life

All participants reported that women from CALD backgrounds experienced substantial barriers to health care and difficulties in everyday prison life, particularly isolation, difficulty in adjusting to prison life and loss of autonomy. These barriers were greatly increased when women had trouble communicating in English and even more profound if there were no other women who spoke the same language at their prison. Some women perceived racism and discriminatory treatment from staff to be the main cause of disrupted health care, such as cancelled appointments and delayed investigations.

Social networks of women from the same language group increased the availability of peers for interpreting, knowledge sharing and support. Informal networks had developed so that women sought medications such as creams or paracetamol from other women, rather than go to the health service themselves. Receiving treatment and medications from health staff could be seen as a victory for the group.

All Western background people, when they come up [to the clinic], they get help in a very like cheerful manner, but it's like when Vietnamese people come up to ask for help, it's always an issue. [Crying]…If by any chance they were given medication, they are always very excited when they come back, just like some achievement or something. They feel like people don't want to help us.(Woman participant 3 translated)

English classes were useful for some women as a way of improving their communication and occupying time in prison. However, for others, it was difficult and stressful.

### 3.2 Health Communication and the use of interpreters

Both women and nurses reported that communication difficulties were the most significant barrier to health care for CALD women in prison.

#### 3.2.1 Deciding on interpreter use

Some nurses felt that formal interpreter services were underutilized. These staff frequently used interpreters and valued them for their ability to improve ease of communication and offer confidentiality for the woman, while also improving the therapeutic relationship because they regarded arranging one as an act of respect. A few nurses did not perceive any significant communication problems and did not use interpreters because they felt they largely dealt with simple health issues for which limited English sufficed.

I think unless there is a formal interview, then you don't worry too much about the interpreter, because you will get across—they'll get across whether it's a cold, or it's a sore ear, or sore throat. I don't have a problem language wise.(Nurse 1)

Most women felt that a formal interpreter should be offered for all significant health interactions, unless the complaint was minor, such as a common cold.

Get a real interpreter. They need to just ring up the interpreter line. It's not that hard to do it. It saves a lot—they don't realise how much drama they can cause somebody.(Woman participant 10—peer Interpreter)

For important issues, our own issues, then you still need an [formal] interpreter especially. But let's say to go up here to ask something there to ask something it's standard then you can get friends to help.(Focus group 1—*translated by professional transcription service*)

Some women reported no problems accessing formal interpreters in prison. However, a larger number reported they were not offered a formal interpreter and were unaware they could request one or felt unable to as they did not wish to seem demanding. Some women felt their English proficiency had been overestimated by health‐care providers, especially during times of stress and illness when their language abilities were further impaired.

Determining the need for interpretation was not always straightforward. Some nurses had experienced women declining an interpreter or indicating understanding during interactions, yet it was unclear if this represented further misunderstanding or a desire to be agreeable.

I've asked them do they want an interpreter…and they say “No” and I will often check in and say, “Do you understand what I'm saying?” and they nod and they smile…but as you're talking to them I wonder whether they can.(Nurse 9)

One peer interpreter confirmed that women often said they understood when they did not.

They always just go, “Yeah yeah yeah yeah yeah,” but they really don't understand. I know they don't understand.(Woman participant 7—peer interpreter)

One woman described how her past affected how she communicated with health staff, feeling that she could not admit that she did not understand.

I have problem not from them, from outside life. When somebody talk to me with loud voice I don't know, I feel my head freeze and like I just [nods] my head—to make them happy and I don't care if I get it or not.(Woman participant 6)

The women reported they were dependent on staff willingness and ability to overcome communication barriers. At times, expectations of poor communication, as well as a fear of being seen to be troublesome, meant women avoided the health service altogether.

Because they're not understanding me, and I am not understanding what they want and then everyone is frustrated and then ah, (gestures with hands). She not ask me and I stopped asking because it's so unnerving and then they hate you more because you ask too much.(Woman participant 13)

#### 3.2.2 Using formal interpreters

Some participants, both women and nurses, expressed ambivalence about formal interpreters. There was particular concern that formal interpreters were not always accurate, including in their medical interpretation.

Sometimes it's [formal interpretation] better, yes, of course. Because I cannot all the time explain correctly what is happening with me. About the interpreter—sometimes she not know in English and sometimes she not know what I mean. So sometimes it's very difficult.(Woman participant 13)

I find sometimes the interpreter, they don't come across as being medically trained. So sometimes it can be a bit difficult using them and it's not—I'm not a fan of using them. I, sort of, put it off, you know. (Nurse 4)

Some nurses distrusted formal interpreters due to concerns about breaches of security protocols.

If they're on the speaker phone and I don't understand the language, are they breaching security that I'm not aware of?(Nurse 3)

Face‐to‐face interpreters were thought by some to be too expensive and time‐consuming to organize. Unpredictable prison schedules and transfers meant that some nurses and women preferred to expedite the consultation using the resources at hand, rather than wait for a formal interpreter (including telephone interpreters) and risk not having a consultation at all.

#### 3.2.3 Peer Interpreters

Peer interpreters were reported to be preferentially used by prison and health staff. Some women and staff felt that, at times, peer interpreters could be better placed as communication brokers, as they were more likely to know the woman and the prison system and to use language that was adjusted for the woman's level of understanding.

One time this lady—she still got me there but the doctor said, “Oh maybe we should try the phone translator.” But it doesn't really work well because the lady told me that I translate more clearly than the translator…and then I know the history, I know what they know about and I know their health more than the phone translator.(Woman participant 7—peer interpreter)

Some nurses felt that by being able to see non‐verbal aspects of the interpreters’ communication, and by personally knowing the peer interpreter from other interactions within the prison, they were more in control and more confident about the outcome of the consultation.

I just find it hard, unless I'm looking at someone to actually hear what they're saying and the body language as well.(Nurse 2)

Additionally, some women had built relationships in prison and preferred to have a friend interpret and provide moral support, as they found it intimidating to approach staff. Peer interpreters could function as informal peer support workers by following up and supporting the patient after the consultation and saw their roles as including translating patient information booklets and reminding patients about treatment advice that had been given. Because of frequent interactions with health services, some women believed they had developed good health literacy and described being proactive in assisting women with their health issues.

They cry to me every day because I'm so busy in the morning, I've got my class to do. They all say, “please, come, help me to see the doctor, help me to translate.” But…they have to come back, bring the paper and say, “Oh can you explain what the doctor write?…[One patient] was on warfarin and warfarin is a very dangerous medication. And she had to do blood levels, tells you the thickness of your blood. So now I learnt all this because I have to read the instruction to her [laughs]. I had to sit there, translate every single word in Chinese.(Woman participant 7—peer interpreter)

Some peer interpreters viewed their role as a trusted advocate of other women and derived satisfaction from it, assisting women well after the consultation was over. These activities could alleviate boredom and give purpose to some women during their prison stay. Some felt a strong responsibility in their role.

That is the system like that, so it's really bad. Imagine if I'm not here, what they're going to do?(Woman participant 7—peer interpreter)

#### 3.2.4 Perceived challenges with peer interpreters

Some nurses and women of LEP were concerned with the lack of confidentiality of peer interpretation. Additionally, prison dynamics could mean women were vulnerable to private information being used against them, and peer interpretation could be affected by conflicting agendas.

I don't want to like rely on these people that probably sometime they can help, but you don't know… if they like you, they may help, otherwise they make it difficult. Like I realized, I am aware that I'm living in prison now, and then you don't really know like how good or how bad people surrounding you are.(Woman participant 3—*translated by professional interpreter*)

There's a whole undercurrent in this community that we don't understand—we're observers of. And there's a whole hierarchy, and a whole subtext…so I'm afraid if they bring a friend in, I don't understand the sub‐text…If we give them what we think would be the best thing for them, will they be stood over for it? Will they trade it for something else?(Nurse 3)

Some peer interpreters took on the role reluctantly and felt pressure to interpret despite their misgivings about inaccuracies and repercussions from errors. Although aware they could decline to interpret, peer interpreters did not always feel comfortable doing so, perceiving a need to “behave” and to please staff and meet the expectations of their cultural community.

I had already told them—the officers—that I don't speak Vietnamese that well…I'm finding other words to go around it and I'm probably not explaining it right…You are put on the spot and how can you not—say no, I'm not doing it. How do you say that without hurting somebody, and with the inmate, the inmate would treat you differently because you said no. Or they would go and do Chinese Whispers, you know, she's our people and she wouldn't even help us.(Woman participant 1—peer interpreter)

Peer interpreters could also be put in the position of assisting, or choosing not to assist, other women when they did not think their claims were reasonable.

Some of these problems that she's got is actually visible, you can see it, and then there's just some really outright silly ones where I think that's just a bit selfish on her behalf to be asking clinic staff about certain stuff like that.(Woman participant 14—peer interpreter)

They described unease at being caught in conflicts between women, health staff or prison staff, or if they could be seen as complicit if the interpretation involved informing on other women or required interpretation of very personal or bad news.

Nobody wanted to interpret for this lady ‘cause apparently she was a trouble‐maker…Even though I knew she was causing problems I felt bad because nobody wanted to do it for her, so I did it…and it was very private and I didn't want to know that stuff. (Woman participant 1—peer interpreter)

## 4 DISCUSSION

**Figure 1:**
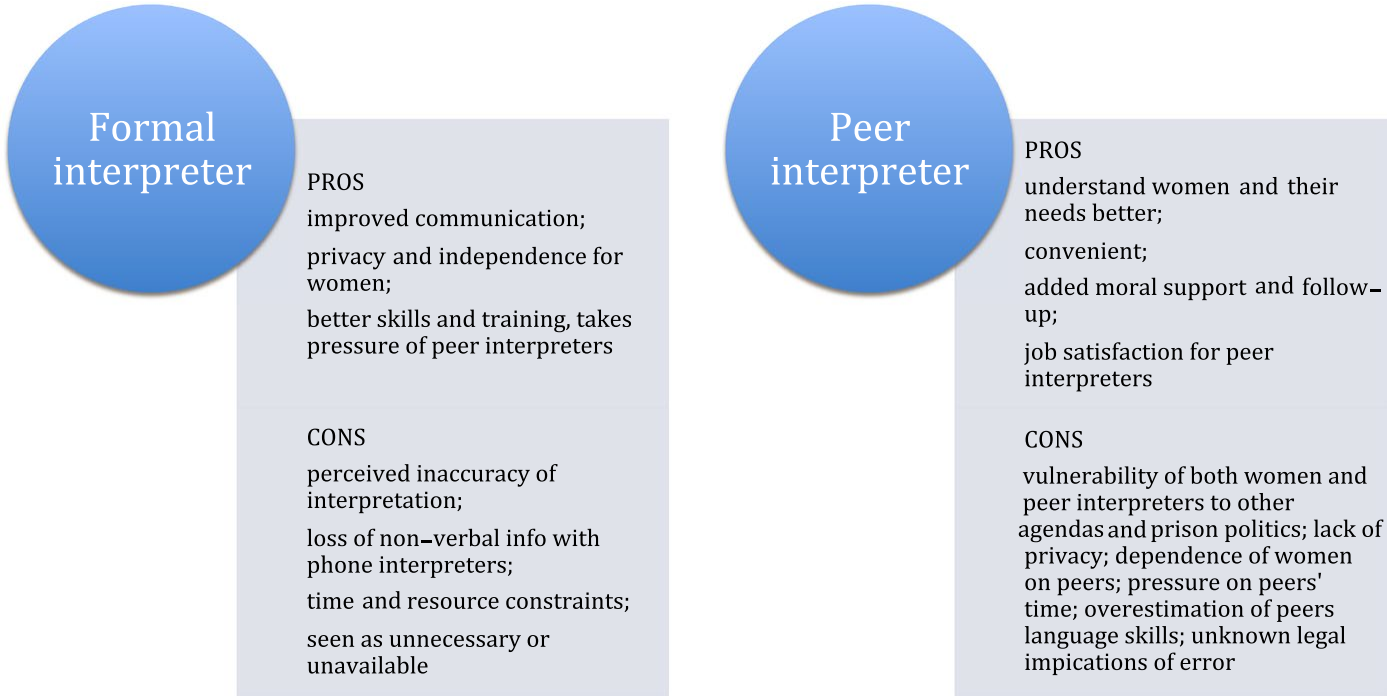
Perceived benefits and challenges of formal and peer interpreters

Women entering prison may suffer shock, fear and disempowerment.^2^ In this study, both the CALD women in prison and their treating nurses found communication across language and culture challenging and that this could potentiate stressful prison experiences and further disempower the women. Interpreters were often not offered in health interactions, and this could be a significant barrier to health‐care access. Failure to offer a formal interpreter for a person with LEP needing health care can constitute a breach of human rights^12^ as well as being clearly outside health service protocols and the evidence base supporting the use of formal interpreters.^16^, ^17^, ^18^ Nevertheless, participants in our study perceived there were both pros and cons of using formal and peer interpreters in the prison health setting (Figure 1). The value, we believe, in recognizing and exploring the issue of informal interpretation in prison is to clarify the risks and benefits and understand the perpetuating factors behind the practice.

Informal interpreters lack training and professional obligations, such as those required by The Australian Institute of Interpreters and Translators code of ethics and standards of practice.^31^ In primary health care outside the prison, the risks of using untrained family members include errors of interpretation due to varying language ability and lack of knowledge of medical terminology, as well as distortion of information due to conflicting roles and agendas of family members as interpreters.^19^, ^20^ Yet, they are commonly used for reasons of personal preference (related to trust, support and advocacy), lack of resources or awareness of resources available.^19^, ^20^

In the prison context, the risks of using untrained peer interpreters are further compounded. Variations from expected professional standards such as accuracy, impartiality, professional role, confidentiality and respect have been seen to occur among non‐professional interpreters in prison settings^15^ and can lead to unintended and negative consequences.^15^ There are few boundaries between the peer interpreter and patient as fellow inmates within a complex prison hierarchy,^8^ where women of LEP are of low status and vulnerable to being “stood over”.^13^ In the forced or “artificial” prison community, women cannot easily seek alternative health care and the consequences of errors of interpretation, conflicting agendas and transgressions of confidentiality, may therefore be greater. These could include physical or emotional harm from others in the prison, disciplinary action or loss of privileges due to (inadvertent or not) security breaches.^32^, ^33^

Additionally, as demonstrated in our research, prisoners may be at risk of being coerced into a peer interpreter role, even if they lacked confidence in their language abilities and felt discomfort in having to interpret private matters or being seen to be complicit in informing on staff or pandering to staff. Decisions to interpret could be clouded by perceived obligations to staff and to their community, and a sense there were no other suitable communication alternatives. This could affect the quality of their interpretation, despite the trust that patients and nurses placed on it.

Our study demonstrated the persistence of peer interpretation was sometimes due to convenience or because formal interpreters were not considered. However, peer interpretation could also be the preferred choice. Some nurses avoided external phone interpreters due to security concerns, despite the Telephone Interpreter Service (TIS) being nationally accredited and approved, with an ongoing quality assurance program.^34^ Issues of quality observed by some nurses and patients were similarly found in the Performance Audit Report of TIS,^35^ suggesting that while they remain the gold standard of interpretation, are not without challenges of their own.

Training health‐care providers in the use of interpreters may improve their understanding of communication difficulties of patients with LEP and their skills in identifying and managing the risks and complexities of such consultations.^21^, ^36^ Our study provides evidence on the need to promote the liberal use of formal interpreters among prison health staff and to inform women they have the right to ask for a formal interpreter. A shared and informed decision‐making process would respect the preferences of the women using such services, while also acknowledging policy and best practice recommendations. This would also promote the autonomy and empowerment of the women should they explicitly choose to utilize a peer interpreter.

Other drivers of peer interpretation in prison appeared to lie in the mediation of trust and in the advocacy they afforded to both staff and patients, particularly as “insiders” of both prison culture and their own cultural background. Cultural capital can be a benefit that at times surpasses the quality of the exchange itself.^20^ In our study, barriers to access were perceived by some to be due to racism and discrimination. For women with a background of trauma and abuse, negative interactions with health‐care providers can have profound emotional impacts and difficulty accessing health care in prison can be interpreted as deliberate blocking of care.^22^ The pre‐existing relationships and rapport the peer interpreters had with both parties may have decreased some barriers to care. Some peer interpreters regarded their role as affirming and satisfying, increasing their motivation to act in this role.

**Figure 2:**
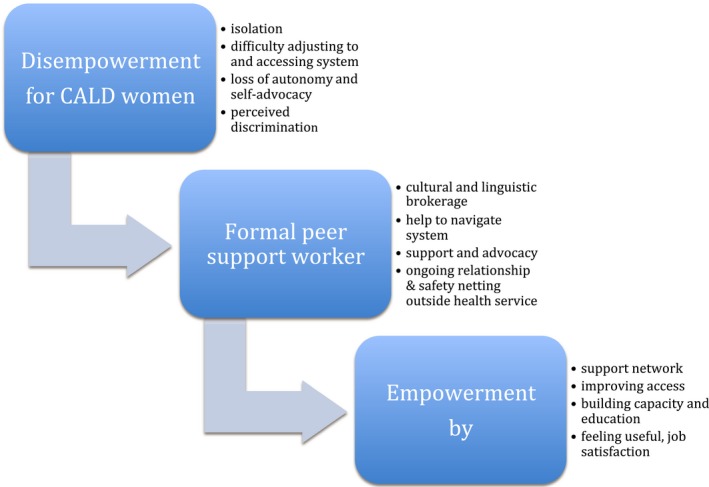
Benefits of peer support worker role

It is apparent that peer interpreters in prison may take on informal roles that are in keeping with peer support workers. Prison peer support programmes are an emerging approach to bridge health service gaps; the research suggests benefits exist, but evidence is generally limited.^37^, ^38^, ^39^ These programmes utilize prisoners who are formally trained and employed in either paid or unpaid roles and include peer support and health education activities.^37^, ^38^, ^39^ They have the potential to reduce barriers to health care and empower CALD women through advocacy and support, while promoting cohesion within the prison community^39^ and supporting its rehabilitative function.^40^ Figure 2 summarizes the formal peer worker role and its potential benefits in the prison setting.

Benefits for the peer support workers in prisons include positive personal growth, satisfaction and improved physical and emotional health; however, benefits are less well defined for recipients of the support.^37^, ^38^, ^41^ It should be noted that the trust and power inherent in peer support worker roles in prison may incur additional security risks, such as distribution of contraband by the peer support worker.^37^, ^38^ Further research is needed.

### 4.1 Limitations

In our study, we aggregated our analysis of women from diverse CALD groups. Although there were strong recurring themes relating to language and cultural difference compared to the general prison population, differences between cultural groups may have emerged with further analysis of larger numbers of participants.

The custodial setting where interviews took place would have limited participants’ ability to respond freely, although they were eager to report both negative and positive experiences. Peer recruitment of the focus groups enabled existing networks of women to participate together. While this meant that existing hierarchies and relationships were reproduced, it also meant that groups were more homogenous in culture and language, potentially reducing the effect of power relationships between cultural and language groups on data collection and increasing comfort and ease of communication among participants.^25^ In addition, individual interviews were conducted where women were able to speak without the constraints of group and community dynamics.

All but one woman declined to have a formal interpreter for their interview, despite our original planning to provide this for everyone. They may have avoided formal interpreters due to lack of trust in prison outsiders and a strong fear of stigma in their external community, from which the formal interpreter may have come,^12^ but this was not explicitly explored during the interview given it was being peer‐interpreted. This may represent some bias towards women who preferred not to use formal interpretation and thus use peers. It could also mean that communication was suboptimal in some individual interviews.

Using a peer interpreter in focus groups has likely influenced our findings on the use of peer interpreters, particularly the discussion on the risks and disadvantages of their use. However, there were also benefits to the women choosing to use them. There was further richness to the data due to her insider understanding of the women's experience in prison, an important consideration with bilingual moderators.^24^ The women already had rapport with the peer interpreter and were more likely to respond openly.^24^ Box 2 provides an example of candour evident among the women, as well as the peer interpreter's ability to translate this. Through purposive sampling and using individual interviews, we were able to canvass countervailing views on the use of peers and explored the views of women for whom peer interpreters did not exist. We also recognized the potential for confirmation bias towards a western point of view associated with translations coming through a peer interpreter who was westernized and potentially more educated,^24^ but this would not have been reduced by the use of formal interpreters.

## 5 CONCLUSIONS

It is essential to overcome communication barriers in order to provide quality health care for CALD women in prison. At times, health‐care providers and women in prison prefer peer interpreters despite best practice recommendations to use formal interpreters. The persistence of their use may be due to their attributes as an informal peer support person and the current failings of prisons to meet the communication needs of women of LEP. However, the peer interpreter role is highly complex for which they are likely to be inadequately skilled, trained or supported. Improved understanding and management of the complexities of communication with both formal and peer interpreters could enable better quality of care and equity of access for CALD women in the prison health service setting.

## ETHICS APPROVAL AND CONSENT TO PARTICIPATE

Ethics approval was sought and was given by the ethics committees of the University of Western Sydney (H10322), the Justice Health & Forensic Mental Health Network (JH&FMHN)(G31/13) and the Department of Corrective Services NSW (13.259026). We obtained written consent, providing the women with consent and participant information forms in English and the woman's language of choice.

## CONFLICT OF INTEREST

PA is a general practitioner and board member with JH&FMHN.
